# In silico design and synthesis of targeted rutin derivatives as xanthine oxidase inhibitors

**DOI:** 10.1186/s13065-019-0585-8

**Published:** 2019-05-23

**Authors:** Neelam Malik, Priyanka Dhiman, Anurag Khatkar

**Affiliations:** 1 Faculty, Department of Pharmaceutical Sciences, M.D. University, Rohtak, 124001 India; 2 Laboratory for Preservation Technology and Enzyme Inhibition Studies, Department of Pharmaceutical Sciences, M.D. University, Rohtak, Haryana India

**Keywords:** Rutin, Xanthine oxidase, Molecular docking, Antioxidant

## Abstract

**Background:**

Xanthine oxidase is an important enzyme of purine catabolism pathway and has been associated directly in pathogenesis of gout and indirectly in many pathological conditions like cancer, diabetes and metabolic syndrome. In this research rutin, a bioactive flavonoid was explored to determine the capability of itself and its derivatives to inhibit xanthine oxidase.

**Objective:**

To develop new xanthine oxidase inhibitors from natural constituents along with antioxidant potential.

**Method:**

In this report, we designed and synthesized rutin derivatives hybridized with hydrazines to form hydrazides and natural acids to form ester linkage with the help of molecular docking. The synthesized compounds were evaluated for their antioxidant and xanthine oxidase inhibitory potential.

**Results:**

The enzyme kinetic studies performed on rutin derivatives showed a potential inhibitory effect on XO ability in competitive manner with IC_50_ value ranging from 04.708 to 19.377 µM and **RU3a**_**3**_ was revealed as most active derivative. Molecular simulation revealed that new rutin derivatives interacted with the amino acid residues PHE798, GLN1194, ARG912, GLN 767, ALA1078 and MET1038 positioned inside the binding site of XO. Results of antioxidant activity revealed that all the derivatives showed very good antioxidant potential.

**Conclusion:**

Taking advantage of molecular docking, this hybridization of two natural constituent could lead to desirable xanthine oxidase inhibitors with improved activity.
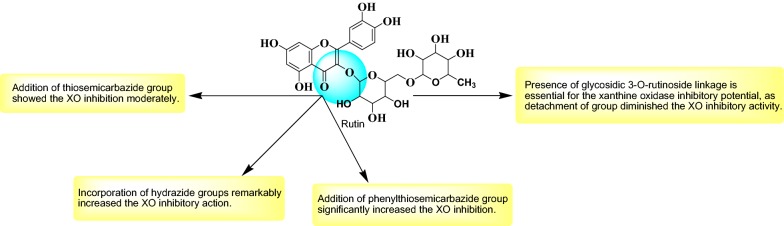

**Electronic supplementary material:**

The online version of this article (10.1186/s13065-019-0585-8) contains supplementary material, which is available to authorized users.

## Introduction

Xanthine oxidase (XO) having molecular weight of around 300 kDa is oxidoreductase enzyme represented in the form of a homodimer. Both the monomers of XO are almost identical and each of them contains three domains namely (a) molybdopterin (Mo-pt) domain at the C-terminal having 4 redox centers where oxidation takes place (b) a flavin adenine dinucleotide (FAD) domain at the centre generally considered as binding site domain and (c) 2[Fe–S]/iron sulfur domain at the N-terminal [1–3]. The catalytic oxidation of XO is two substrates reaction on the xanthine and oxygen at the enzymatic centre. While xanthine undergoes oxidation reaction near to the Mo-pt center/substrate binding domain of XO, simultaneously substrate oxygen undergoes reduction at FAD center and electron transfer takes place leading to formation of superoxide anion (O^2−^) or hydrogen peroxide (H_2_O_2_) free radicals. [4–8]. This catalytic reaction results in formation uric acid as a final product and oxygen reactive species in form of free radicals. The excessive generation of uric acid leads to a condition like hyperuricemia which is a key factor in development of gout [1, 9], and uncontrolled amounts of reactive oxygen species causes many pathological conditions like cardiovascular disorders, inflammatory diseases and hypertensive disorders. Xanthine oxidase (XO; EC 1.17.3.2) has been considered as significantly potent drug target for the cure and management of pathological conditions prevailing due to high levels of uric acid in the blood stream. [10–17]. Considering the above fact, by inhibiting XO selectively could be better treatment plan for disorders caused by XO directly or indirectly including gout, inflammatory disease, oxidative damage and cancer [3, 18, 19]. Generally, XO inhibitors have been categorized into purine and non-purines inhibitors differentiated on the basis of their chemically derived skeleton structure. The first purine derived XO inhibitor discovered and approved by US FDA was Allopurinol as marketed drug for gout and hyperuricemia [20, 21]. Considering the life threatening side effects like Stevens–Johnsons syndrome caused by allopurinol use, scientists turned their interest into non-purine XO inhibitors and an immense accomplishment has been received in this direction with development of new drug Febuxostat [22–25]. This non-purine candidate produced minor and non-life threatening adverse effects in comparison to Allopurinol [26–29]. Extending our previous successful effort to achieve new xanthine oxidase inhibitors from natural sources, in this report we investigated and developed some new rutin derived xanthine oxidase inhibitor [30].

Rutin is a well characterized bioactive plant flavonoid having great therapeutic importance for the treatment of many disease like conditions including cytotoxicity, antioxidant activity, antibacterial property and anti-inflammatory action [31–34]. Due to these pharmacological activities rutin is explored widely and great success have been achieved in order to get drug like candidates.
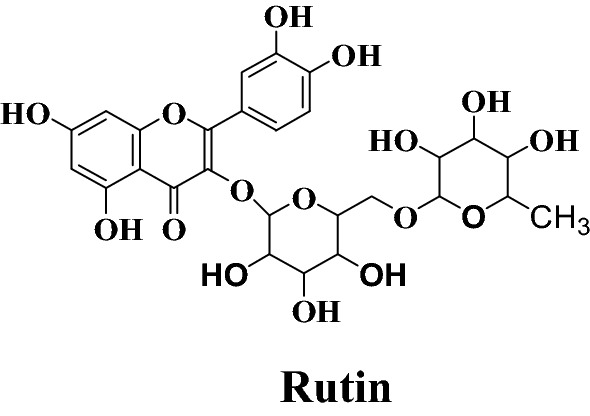


**Fig. 1:**
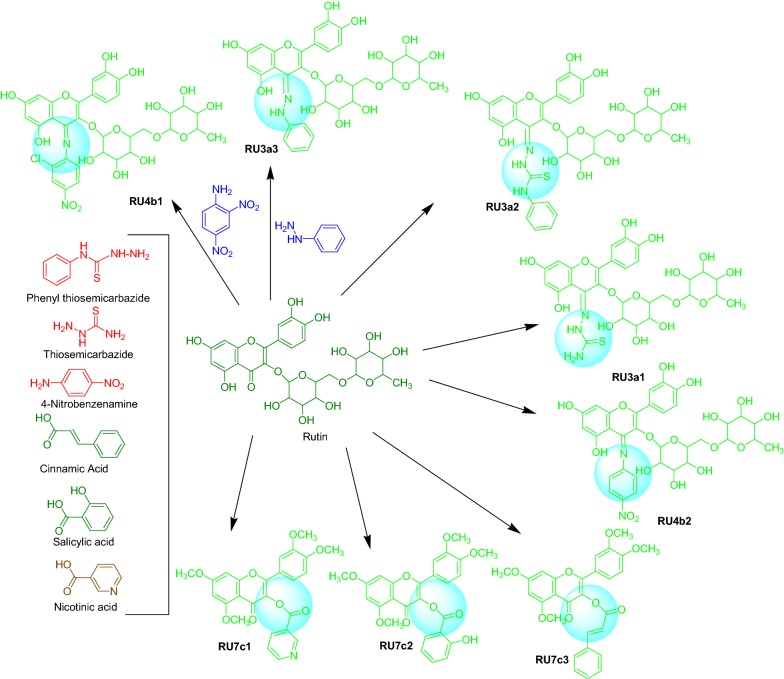
Design strategy for the development of rutin derivatives

Taking advantage of molecular docking techniques new compounds with potential drugability for the targeted enzyme might be achieved with a precise knowledge of mechanism of action. With the combined approach of molecular docking and synthetic chemistry, in this research we developed some new potential compounds against xanthine oxidase (Fig. 1).

## Experimental

### Chemicals and instrumentation

For this research, the analytical grade chemicals necessary for synthesis and antioxidant activity were purchased from Hi-media Laboratories. The in vitro evaluation of the human xanthine oxidase inhibitory activity was performed by measuring hydrogen peroxide (H_2_O_2_) production from oxidation of xanthine oxidase by the substrate xanthine, utilizing the human xanthine oxidase assay kit (Sigma USA). The progress of reaction was observed through thin layer chromatography (TLC) on 0.25 mm precoated silica gel plates purchased from Merck, reaction spots were envisaged in iodine compartment and UV. Melting points were measured using a Sonar melting point apparatus and uncorrected. ^1^H NMR and ^13^C NMR spectra were documented in DMSO and deuterated CDCl_3_ respectively on Bruker Avance II 400 NMR spectrometer at the frequency of 400 MHz using tetramethylsilane standard (downfield) moreover chemical shifts were expressed in ppm (δ) using the residual solvent line as internal standard. Infrared (IR) spectra were recorded on Perkin Elmer FTIR spectrophotometer by utilizing KBr pellets system.

### Molecular docking

In silico docking studies was done with integrated Schrodinger software using Glide module for enzyme ligand docking [35].

#### Protocol followed for docking procedures

##### Preparation of protein

The 3D crystal structure of human xanthine oxidase co-crystalised with salicylic acid was retrieved from Protein Data Bank (PDB ID. 2E1Q). The targeted protein structure was further refined in the Protein Preparation Wizard to obtain the optimized and chemically accurate protein configuration. For that, the co-crystalised enzyme (XO) was retrieved directly from Protein data bank in maestro panel followed by removal of water molecules, addition of H atoms, addition of missing side chains and finally minimization was done to obtain the optimized structure.

##### Preparation of ligand

The 3D-structures of rutin derived compounds to be docked against XO were built in maestro building window. Ligand preparation was performed in Ligprep module.

##### Active site prediction

To predict the binding site/active site Site Map application of glide was utilized. Out of top three active site, the one having larger radius was selected. Validation of binding site was done by redocking the salicylic acid and RMSD value was observed. RMSD value of less than 0.2 validated the docking procedure and active site was defined for docking of new rutin analogs.

##### Glide docking

To carry out docking, Firstly the receptor grid generation tool was utilized to around the active/binding site of xanthine oxidase and glide docking with extra precision was used to visualize the interaction of protein and ligand. The top active ligand was selected for wet lab synthesis and evaluation of pharmacological activity.

### Synthetic procedures

#### Procedures for synthesis of rutin derivatives (Scheme 1)

(A) *General procedure for synthesis of hydrazine derivatives RU3a*_*(1*– *4)*_
  0.001 mol of rutin was taken in round bottom flask and dissolved in 50 ml of ethanol. Different hydrazines (0.001 mol) were added to the flask and reaction mixture was refluxed for 5–6 h at 40 °C. Completion of reaction was monitored by TLC. The product thus obtained was filtered and filtrate was concentrated to obtain the final product. The final product was recrystallised to obtain the pure compound.
(B) *General procedure for synthesis of anilline derivatives RU4b*_*(1*– *2)*_
  0.001 mol of the intermediate obtained above was taken in round bottom flask and dissolved in 50 ml of ethanol. Different anillines (0.001 mol) were added to the flask and reaction mixture was refluxed for 8–10 h at 40 °C. Completion of reaction was monitored by TLC. The product thus obtained was filtered and filtrate was concentrated to obtain the final product. The final product was recrystallised to obtain the pure compound.
(C) *General procedure for synthesis of methylated rutin derivatives RU7c*_*(1*– *3)*_
  Rutin was methylated by methyl sulphate in presence of potassium carbonate and dimethyl formamide by stirring along with reflux at 40 °C for 48 h to generate tetramethylated rutin. Acidolysis of above was done to obtain the intermediate compound (RUI) by refluxing it with HCl and 95% ethanol for 4 h. The intermediate compound (RUI) was then refluxed with different phenolic acid to obtain their ester derivatives.

**Scheme 1:**
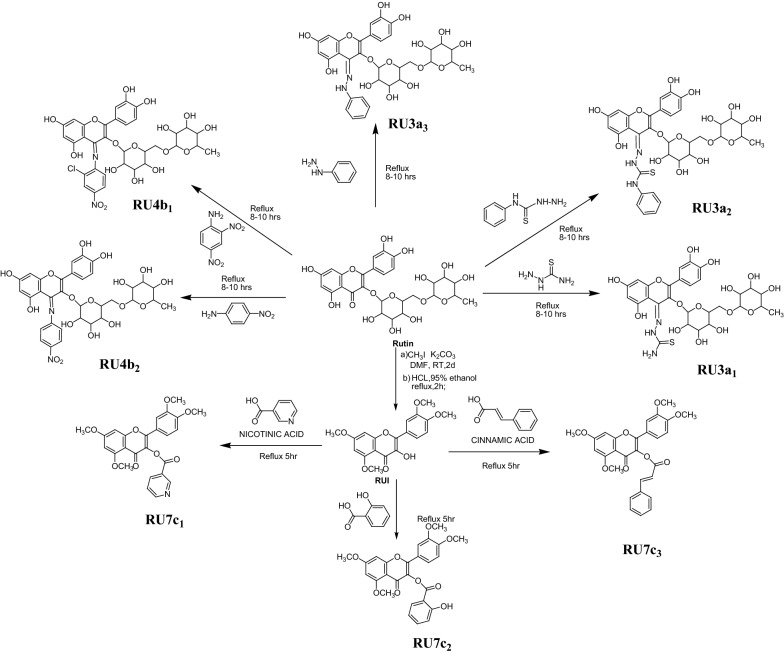
Synthesis of rutin derivatives

##### Spectral data

RU3a_1_ yield 69.6% R_f_ 0.6 [Mobile Phase for TLC—Methanol:Glacial acetic acid:Formic acid:Water (3:2.9:0.8:0.5)] M.pt. (231–232) IR (KBR pellets) cm^−1^ 1) 3222 (O–H str., Ar), 1609 (C=N str.), 1501 (C=C str.), 1206 (O–CH_3_), 1128 (C=S Str.) ^1^H NMR (400 MHz, DMSO-*d*_6_) δ 7.81 (dd, *J* = 7.5, 1.5 Hz, 1H), 7.59 (d, *J* = 1.5 Hz, 1H), 6.82 (d, *J* = 7.5 Hz, 1H), 6.48 (dd, *J* = 15.0, 1.5 Hz, 2H), 6.28 (t, *J* = 7.0 Hz, 1H), 4.13 (t, *J* = 7.0 Hz, 1H), 3.89–3.81 (m, 3H), 3.71 (dd, *J* = 12.4, 6.9 Hz, 1H), 3.67–3.54 (m, 3H), 2.32 (dt, *J* = 12.4, 7.0 Hz, 1H), 2.28–2.16 (m, 2H), 2.06–2.04 (m, 1H), 1.97–1.92 (m, 2H), 1.74–1.66 (m, 2H). ^13^C NMR (100 MHz, Chloroform-*d*) δ 180.16, 163.73, 155.81, 154.70, 152.34, 148.70, 145.50, 133.79, 133.45, 120.73, 120.41, 115.79, 115.09, 102.38, 99.59, 99.00, 91.11, 80.48, 73.58, 73.26, 72.40, 71.83 (d, *J* = 10.5 Hz), 66.02, 40.22, 37.43, 28.26, 26.90. m/z found for C_28_H_33_N_3_O_15_S: 683 (M^+^) 687 (M + 1)^+^. Anal calcd for C_28_H_33_N_3_O_15_S: C, 52.91; H, 5.23; N, 6.61; O, 35.20; S, 5.04 Found: C, 52.93; H, 5.21; N, 6.60; O, 35.19; S, 5.06.

RU3a_2_ yield 72.5% R_f_ 0.7 [Mobile Phase for TLC—Methanol:Glacial acetic acid:Formic acid:Water (3:2.9:0.8:0.5)] M.pt. (255–257) IR (KBR pellets) cm^−1^) 3468 (O–H str., Ar), 1639 (C=N str.), 1596 (C=C str.), 1218 (O–CH_3_), 1150 (C=S Str.) ^1^H NMR (400 MHz, DMSO-*d*_6_) δ 7.78–7.60 (m, 3H), 7.49 (d, *J* = 1.5 Hz, 1H), 7.39–7.29 (m, 2H), 7.10–7.01 (m, 1H), 6.86 (d, *J* = 7.5 Hz, 1H), 6.52 (dd, *J* = 15.0, 1.5 Hz, 2H), 6.24 (t, *J* = 7.0 Hz, 1H), 4.04 (t, *J* = 7.0 Hz, 1H), 3.98–3.88 (m, 3H), 3.78 (dd, *J* = 12.4, 6.9 Hz, 1H), 3.68–3.64 (m, 3H), 2.28 (dt, *J* = 12.4, 7.0 Hz, 1H), 2.14–2.11 (m, 2H), 2.09–2.06 (m, 1H), 1.87–1.84 (m, 2H), 1.74–1.71 (m, 2H). ^13^C NMR (100 MHz, Chloroform-*d*) δ 174.93, 164.50, 160.96, 155.78, 150.30, 148.16, 145.55, 139.23, 130.44, 128.67, 124.46, 123.85, 123.09, 122.39, 121.81, 116.06, 115.83, 103.40, 99.09, 97.71, 95.05, 82.37, 73.06 (d, *J* = 19.1 Hz), 72.87 (d, *J* = 12.2 Hz), 72.47, 72.35, 71.92, 65.19, 41.10, 38.86, 29.40, 27.86. m/z found for C_34_H_37_N_3_O_15_S: 759 (M^+^) 760 (M + 1)^+^. Anal calcd for C_34_H_37_N_3_O_15_S: C, 53.75; H, 4.91; N, 5.53; O, 31.59; S, 4.22. Found: C, C, 53.77; H, 4.93; N, 5.56; O, 31.59; S, 4.24.

RUT3a_3_ yield 61% R_f_ 0.6 [Mobile Phase for TLC—Methanol:Glacial acetic acid:Formic acid:Water (3:2.9:0.8:0.5)] M.pt. (235–237) IR (KBR pellets) cm^−1^) 3475 (O–H str., Ar), 1641 (C=N str.), 1580 (C=C str.), 1220 (O–CH_3_), 1155 (C=S Str.) ^1^H NMR (400 MHz, DMSO-*d*_6_) δ 7.70 (dd, *J* = 7.5, 1.5 Hz, 1H), 7.56 (d, *J* = 1.5 Hz, 1H), 7.46–7.38 (m, 2H), 7.32–7.23 (m, 2H), 7.07–6.98 (m, 1H), 6.89 (d, *J* = 7.5 Hz, 1H), 6.35 (dd, *J* = 15.0, 1.5 Hz, 2H), 6.19 (t, *J* = 7.0 Hz, 1H), 4.09 (t, *J* = 7.0 Hz, 1H), 4.02–3.88 (m, 3H), 3.68 (dd, *J* = 12.4, 6.9 Hz, 1H), 3.66–3.54 (m, 3H), 2.33 (dt, *J* = 12.4, 7.0 Hz, 1H), 2.21–2.19 (m, 2H), 1.96–1.88 (m, 2H), 1.87–1.85 (m, 2H) (Additional file 1). ^13^C NMR (100 MHz, Chloroform-*d*) δ 164.50, 160.96, 155.78, 150.30, 148.16, 145.55, 143.60, 132.14, 129.50, 124.46, 122.39, 121.81, 121.19, 118.32, 116.06, 115.83, 104.75, 94.15, 93.97, 91.01, 83.98, 79.41 (d, *J* = 19.1 Hz), 78.77 (d, *J* = 12.2 Hz), 77.09, 73.82, 68.48, 42.85, 37.51, 23.82, 23.17. m/z found for C_33_H_36_N_2_O_15_: 700 (M^+^) 701 (M + 1)^+^. Anal calcd for C_33_H_36_N_2_O_15_: C, 56.57; H, 5.18; N, 4.00; O, 34.25. Found: C, 56.58; H, 5.20; N, 4.00; O, 34.27.

RU4b_1_ yield 74.3% R_f_ 0.6 [Mobile Phase for TLC—Methanol:Glacial acetic acid:Formic acid:Water (3:2.9:0.8:0.5)] M.pt. (259–260) IR (KBR pellets) cm^−1^ 1) 1725 (C=O str.), 1631 (C=N str.), 1603 (C=C str.), 1234 (O–CH_3_), 1268 (C–O str., ester) ^1^H NMR (400 MHz, DMSO-*d*_6_) δ 8.38 (d, *J* = 1.5 Hz, 1H), 8.15 (dd, *J* = 7.5, 1.5 Hz, 1H), 7.69 (dd, *J* = 7.5, 1.5 Hz, 1H), 7.2 (d, *J* = 1.5 Hz, 1H), 7.40 (d, *J* = 7.5 Hz, 1H), 6.81 (d, *J* = 7.5 Hz, 1H), 6.47 (dd, *J* = 10.8, 1.5 Hz, 2H), 6.22 (t, *J* = 7.0 Hz, 1H), 4.11 (t, *J* = 7.0 Hz, 1H), 3.98–3.90 (m, 3H), 3.79 (dd, *J* = 12.4, 6.9 Hz, 1H), 3.71–3.61 (m, 3H), 2.42 (dt, *J* = 12.4, 7.0 Hz, 1H), 2.39– 2.31 (m, 2H), 2.29–2.28 (m, 1H), 1.87–1.77 (m, 2H). ^13^C NMR (100 MHz, Chloroform-*d*) δ 169.14, 168.95, 168.11, 166.86, 150.94, 144.52, 144.24, 142.37, 140.47, 131.18, 128.56, 125.41, 123.81, 122.54 (d, *J* = 14.8 Hz), 121.81, 113.64, 113.17, 106.71, 97.09, 96.89, 93.98, 82.37, 75.79 (d, *J* = 19.1 Hz), 73.17 (d, *J* = 12.2 Hz), 73.06, 72.69, 71.01, 65.19, 41.10, 38.86, 28.85, 27.44. m/z found for H_33_ClN_2_O_17_: 764 (M^+^) 766 (M + 2)^+^. Anal calcd for C_33_H_33_ClN_2_O_17_: C, 51.81; H, 4.35; Cl, 4.63; N, 3.66; O, 35.55. Found: C, 51.83; H, 4.36; Cl, 4.65; N, 3.64; O, 35.53.

RU4b_2_ yield 83.5% R_f_ 0.8 [Mobile Phase for TLC—Methanol:Glacial acetic acid:Formic acid:Water (3:2.9:0.8:0.5)] M.pt. (253–254) IR (KBR pellets) cm^−1^ 1) 1785 (C=O str.), 1637 (C=N str.), 1561 (C=C str.), 1258 (O–CH_3_), 1234 (C–O str., ester) ^1^H NMR (400 MHz, DMSO-*d*_6_) δ 8.21–8.14 (m, 2H), 7.79 (dd, *J* = 7.5, 1.5 Hz, 1H), 7.59 (d, *J* = 1.5 Hz, 1H), 7.32–7.25 (m, 2H), 6.75 (d, *J* = 7.5 Hz, 1H), 6.44 (dd, *J* = 14.1, 1.5 Hz, 2H), 6.27 (t, *J* = 7.0 Hz, 1H), 4.15 (t, *J* = 7.0 Hz, 1H), 3.98–3.95 (m, 3H), 3.88 (dd, *J* = 12.4, 6.9 Hz, 1H), 3.67–3.55 (m, 3H), 2.22 (dt, *J* = 12.4, 7.0 Hz, 1H), 2.14–2.11 (m, 2H), 2.09–2.06 (m, 1H), 1.76–1.73 (m, 2H), 1.67–1.55 (m, 2H).

^13^C NMR (100 MHz, Chloroform-*d*) δ 173.89, 164.58, 163.50, 158.34, 152.36, 151.92, 148.16, 146.53, 145.55, 128.56, 125.27, 124.36, 122.39, 121.81, 116.06, 115.83, 108.81, 93.06, 97.81, 90.53, 82.19, 73.80 (d, *J* = 19.1 Hz), 72.67 (d, *J* = 12.2 Hz), 72.36, 72.12, 71.08, 64.86, 42.81, 36.15, 28.55, 26.98. m/z found for C_33_H_34_N_2_O_17_:730 (M^+^) 731 (M + 1)^+^. Anal calcd for C_33_H_34_N_2_O_17_: C, 54.25; H, 4.69; N, 3.83; O, 37.23. Found: C, 54.27; H, 4.70; N, 3.85; O, 37.25.

RU7C_1_ yield 83.5% R_f_ 0.8 [Mobile Phase for TLC—Methanol:Glacial acetic acid:Formic acid:Water (3:2.9:0.8:0.5)] M.pt. (189–190) IR (KBR pellets) cm^−1^ 1) 1715 (C=O str.), 1627 (C=N str.), 1607 (C=C str.), 1234 (O–CH_3_), 11,944 (C–O str., ester) ^1^H NMR (400 MHz, DMSO-*d*_6_) δ 9.11 (d, *J* = 1.5 Hz, 1H), 8.77–8.70 (m, 1H), 8.14 (dt, *J* = 7.5, 1.5 Hz, 1H), 7.92 (dd, *J* = 7.5, 1.5 Hz, 1H), 7.68 (d, *J* = 1.5 Hz, 1H), 7.51 (t, *J* = 7.5 Hz, 1H), 6.93–6.83 (m, 2H), 6.23 (d, *J* = 1.5 Hz, 1H), 3.92 (s, 3H), 3.83 (d, *J* = 0.9 Hz, 6H), 3.76 (s, 3H). ^13^C NMR (100 MHz, Chloroform-*d*) δ 174.99, 164.48, 164.18, 160.33, 157.96, 156.60, 153.53, 151.74, 150.80, 149.32, 138.25, 128.95, 123.72, 123.22, 122.87, 122.65, 113.70, 112.82, 107.81, 95.68, 93.25, 56.20, 55.88 (d, *J* = 2.6 Hz), 55.62. m/z found for C_25_H_21_NO_8_:463 (M^+^) 464 (M + 1)^+^. Anal calcd for C_25_H_21_NO_8_: C, 64.79; H, 4.57; N, 3.02; O, 27.62. Found: C, 64.80; H, 4.58; N, 3.00; O, 27.60.

RU7C_2_ yield 62.5% R_f_ 0.6 [Mobile Phase for TLC—Methanol:Glacial acetic acid:Formic acid:Water (3:2.9:0.8:0.5)] M.pt. (186–188) IR (KBR pellets) cm^−1^ 1) 1764 (C=O str.), 1619 (C=N str.), 1595 (C=C str.), 1277 (O–CH_3_), 1214 (C–O str., ester) ^1^H NMR (400 MHz, DMSO-*d*_6_) δ 7.91 (ddd, *J* = 7.5, 6.5, 1.5 Hz, 2H), 7.67 (d, *J* = 1.5 Hz, 1H), 7.47 (td, *J* = 7.5, 1.5 Hz, 1H), 7.09 (td, *J* = 7.5, 1.5 Hz, 1H), 6.97–6.88 (m, 2H), 6.86 (d, *J* = 1.5 Hz, 1H), 6.28 (d, *J* = 1.5 Hz, 1H), 3.97 (s, 3H), 3.80 (d, *J* = 0.7 Hz, 6H), 3.67 (s, 3H). ^13^C NMR (100 MHz, Chloroform-*d*) δ 171.85, 168.95, 167.67, 165.22, 158.95, 157.67, 148.53, 146.92, 133.72, 131.16, 128.84, 124.78, 124.78, 123.22, 122.87, 116.52, 113.70, 108.53, 104.92, 92.81, 90.38, 53.06, 52.81, 52.76 (d, *J* = 2.6 Hz), 51.65. m/z found for C_26_H_22_O_9_:478 (M^+^) 479 (M + 1)^+^. Anal calcd for C_26_H_22_O_9_: C, 65.27; H, 4.63; O, 30.10. Found: C, 65.27; H, 4.63; O, 30.10.

RU7C_3_ yield 71% R_f_ 0.7 [Mobile Phase for TLC—Methanol:Glacial acetic acid:Formic acid:Water (3:2.9:0.8:0.5)] M.pt. (165–166) IR (KBR pellets) cm^−1^ 1) 1710 (C=O str.), 1637 (C=N str.), 1596 (C=C str.), 1258 (O–CH_3_), 1194 (C–O str., ester) ^1^H NMR (400 MHz, DMSO-*d*_6_) δ 7.98 (dd, *J* = 7.5, 1.5 Hz, 1H), 7.76 (d, *J* = 1.5 Hz, 1H), 7.30–7.20 (m, 5H), 6.91–6.86 (m, 2H), 6.23 (d, *J* = 1.5 Hz, 1H), 3.93 (s, 3H), 3.88 (d, *J* = 0.9 Hz, 6H), 3.69 (s, 3H), 2.93–2.84 (m, 2H), 2.73 (td, *J* = 7.0, 0.8 Hz, 2H). ^13^C NMR (100 MHz, Chloroform-*d*) δ 175.20, 170.26, 164.48, 160.33, 157.96, 156.95, 150.80, 149.32, 139.89, 128.47–128.31 (m), 126.14, 123.22, 122.87, 113.70, 112.82, 107.81, 99.41, 98.77, 53.17, 53.06 (d, *J* = 2.6 Hz), 52.69, 51.86, 34.56, 30.26. m/z found for C_28_H_24_O_8_:488 (M^+^) 489 (M + 1)^+^. Anal calcd for C_28_H_24_O_8_: C, 68.85; H, 4.95; O, 26.20. Found: C, 68.87; H, 4.90; O, 26.20.

### Evaluation of biological activity

#### In vitro evaluation of xanthine oxidase inhibitory activity

The method opted to evaluate the inhibitory potential of rutin derivatives was a modified protocol of Sigma, done by UV-spectrophotometric method by using xanthine oxidase activity assay kit purchased from sigma (MAK078, sigma-aldrich.co, USA). The colorimetric product obtained in the form of hydrogen peroxide generated during the oxidation of XO was determined by a coupled enzyme technique, measured at 570 nm in a 96-well plate, using the plate reader EPOCH™ “MICROPLATE READER (BIOTEK).one unit of XO is defined as the amount of enzyme that catalyzes the oxidation of xanthine substrate, yielding 1.0 µmol of uric acid and hydrogen peroxide per minute at 25 °C. Reagents used were 44 µL of xanthine oxidase assay buffer, 2 µl xanthine substrate solution and 2 µl of Xanthine Oxidase enzyme solution. All the solutions mentioned above were mixed to prepare reaction mixture. The different concentrations of synthesized derivatives having final volume 50 µl were prepared in dimethyl sulfoxide (DMSO) and added to 96 well plate. To each well 50 µl of reaction mix was added and mixed well. After 2–3 min initial measurement was taken. The plates were incubated at 25 °C taking measurements at every 5 min. Allopurinol served as positive control. Absorbance at different time intervals was noted for further statistical analysis.

#### In vitro evaluation of antioxidant activity by DPPH method

The antioxidant potential of rutin derivatives was performed by DPPH method evaluated in the form of IC_50_ estimated using the ELISA plate reader EPOCH™ “MICROPLATE READER (BIOTEK). This method opted for evaluation of free radical scavenging activity of DPPH was based on modified procedure described by Dhiman et al. [36]. The tested compounds were prepared in methanolic solution and reacted with methanolic solution of DPPH at 37 °C. The reaction mixture was prepared in 96-well plate by adding 50 µL of sample, 50 µl of methanol and 50 µl of DPPH solution prepared in 0.1 mM methanol. The mechanism of action of DPPH assay was based on the fact that DPPH radical get reduced during its reaction with an antioxidant compound and results in changes of color (from deep violet to light yellow). The absorbance was read at 517 nm for 30 min at an interval of 5 min of using ELISA microplate reader. The mixture of methanol (5.0 ml) and tested compounds (0.2 ml) serve as blank. Ascorbic acid served as positive control.

#### Hydrogen peroxide scavenging (H_2_O_2_) assay

To compare and best evaluate the antioxidant potential of newly synthesized rutin derivatives, hydrogen peroxide assay was performed by the method described by Patel et al. [37] with some modifications. The solution of H_2_O_2_ (100 mM) was prepared via adding up different concentrations of synthesized derivatives ranging from 5 to 80 μg/ml to H_2_O_2_ solution (2 ml), prepared in 20 mM phosphate buffer of pH 7.4. Finally, the absorbance of H_2_O_2_ was measured at 230 nm after incubating for 10 min next to a blank reading of phosphate buffer without H_2_O_2_. For every measurement, a fresh reading of blank was taken to carry out background correction. For control sample containing H_2_O_2_ was scanned for absorbance at 230 nm. Results calculated as percentage of hydrogen peroxide inhibition was estimated by the formula [(A_b_–A_t_)/A_0_] × 100, where A_b_ is the absorbance of the control and A_t_ is the absorbance of compounds/standard taken as l-ascorbic acid (5–80 μg/ml) are shown in Table 5.

#### ADMET studies

The pharmacokinetic and pharmacological parameters of newly synthesized compounds were predicted with the help of Schrodinger suite. In-silico ADMET-related properties were computed using Qikprop application of Schrodinger software (Table 1). QikProp program generates set of physicochemically significant descriptors which further evaluates ADMET properties. The whole ADME-compliance score-drug-likeness parameter is used to predict the pharmacokinetic profiles of the ligands. This parameter determines the number of property descriptors calculated via QikProp which fall outside from the optimum range of values for 95% of noted drugs. Initially, all compound structures were neutralized before operated through Qikprop. The neutralizing step is crucial, as QikProp is unable to neutralize ligands in normal mode. Qikprop predicts both pharmacokinetically significant properties and physicochemically significant descriptors. It application run in normal mode which predicted IC_50_ value for blockage of HERG K + channels (log HERG), predicted apparent Caco-2 cell permeability in nm/s (QPPCaco), brain/blood partition coefficient (QPlogBB), predicted skin permeability (QPlogKp), prediction of binding to human serum albumin (QPlogKhsa) and predicted apparent Madin–Darby Canine Kidney (MDCK) cell permeability in nm/s (QPPMDCK). Solubility of drug was predicted as octanol/water partition coefficient (QPlogPo/w). Aqueous solubility of compound defined in terms of log S (S in mol dm^−3^) is the concentration of the solute in a saturated solution that is in equilibrium with the crystalline solid.

**Table 1:** ADMET data of natural ligands calculated using Qik Prop simulation

Compound	QPlogPo/w	QPlogS	QPlogHERG	QPPCaco	QPlogBB	QPPMDCK	QPlogKp	QPlogKhsa	Human oral absorption	Percent human oral absorption
RU3a_1_	− 1.084	− 3.257	− 5.488	511.672	− 2.173	625.905	− 6.818	− 0.902	2	81
RU3a_2_	0.866	− 4.593	− 7.183	605.947	− 1.139	853.322	− 4.846	− 0.635	2	77
RU3a_3_	0.444	− 2.809	− 5.496	758.912	− 1.381	793.01	− 4.796	− 0.58	3	76
RU4b_1_	− 0.044	− 3.745	− 6.548	563.916	− 2.192	641.237	− 5.52	− 0.747	1	60
RU4b_2_	0.407	− 4.15	− 6.511	941.594	− 2.757	730.468	− 6.278	− 0.533	1	50
RU7c_1_	3.322	− 4.469	− 6.334	1460.431	− 0.726	744.963	− 1.477	− 0.218	3	100
RU7c_2_	4.878	− 5.717	− 6.59	2335.951	− 0.63	1237.701	− 0.774	0.383	3	100
RU7c_3_	− 0.334	− 3.885	− 6.168	743.251	− 1.271	971.012	− 6.276	− 0.735	2	50
Rutin	− 0.28	− 2.94	− 5.166	827.655	− 3.378	682.554	− 5.639	− 0.703	1	30
Allopurinol	− 1.365	− 2.932	− 0.839	569.551	− 3.6	− 570.702	− 6.890	− 0.986	2	50

Descriptor standard range: QPlogPo/w, − 2.0 to 6.5; QPlogS, − 6.5 to 0.5; QPlogHERG, concern below –5; QPPCaco, < 25 poor, > 500 great; QPlogBB, − 3.0 to 1.2; QPPMDCK, < 25 poor, > 500 great; QPlogKp, − 8.0 to − 1.0; QPlogKhsa, − 1.5 to 1.5; human oral absorption, 1, 2, or 3 for low, medium, or high; percent human oral absorption, > 80% is high

## Result and discussion

### Molecular docking

To rationalize the structure activity relationship observed in this research and to foreknow the potential interaction of the synthesized compounds with XO, molecular simulation studies were carried out using Schrödinger suite (Schrödinger Release 2018-2, Schrödinger, LLC, New York, NY, 2018).The crystal structure of xanthine oxidase with PDB code 2E1Q was adopted for the docking calculations. Based on the docking score and binding energy calculation, top ranking derivatives were established and compared with the IC_50_ calculated from in vitro activity (Table 2). Important interactions were depicted as hydrophobic regions, hydrogen bonding, polar interactions and pi–pi bonding visualized in the active pocket of xanthine oxidase revealed through Site map application of Schrodinger suite. The derivatives having better docking scores than rutin were kept for further synthetic procedures and the remaining were discarded. To observe the binding interaction in detail, 3D poses of two most active compounds RU3a_3_ and RU3a_1_ were visualized and compared with native rutin and standard drug Allopurinol. The residues of binding pocket involved in the interaction were reported as GLN 1194, ARG912, MET1038, GLN1040, PHE798 and SER1080. Similar binding cavity was observed by Li et al. during the docking analysis of newly synthesized non-purine XO inhibitors [38].

**Table 2:** Comparison of in vitro activity and molecular docking studies

Compound	Docking score	Binding energy [ΔG (KJ/mol)]	IC_50_ (µM)
RU3a_1_	− 12.907	− 88.383	09.924 ± 0.01
RU3a_2_	− 11.456	− 67.673	07.905 ± 0.15
RU3a_3_	− 13.244	− 91.242	04.870 ± 0.02
RU4b_1_	− 11.591	− 60.323	15.037 ± 0.01
RU4b_2_	− 12.021	− 72.991	12.541 ± 0.45
RU7c_1_	− 11.310	− 55.854	19.377 ± 0.38
RU7c_2_	− 10.980	− 61.268	17.428 ± 0.01
RU7c_3_	11.037	50.217	13.476 ± 0.25
Rutin	− 10.944	− 45.549	20.867 ± 0.12
Allopurinol	− *3.366*	− *17.231*	*10.410* *±* *0.72*

Italic values indicating standard drug

**Fig. 2:**
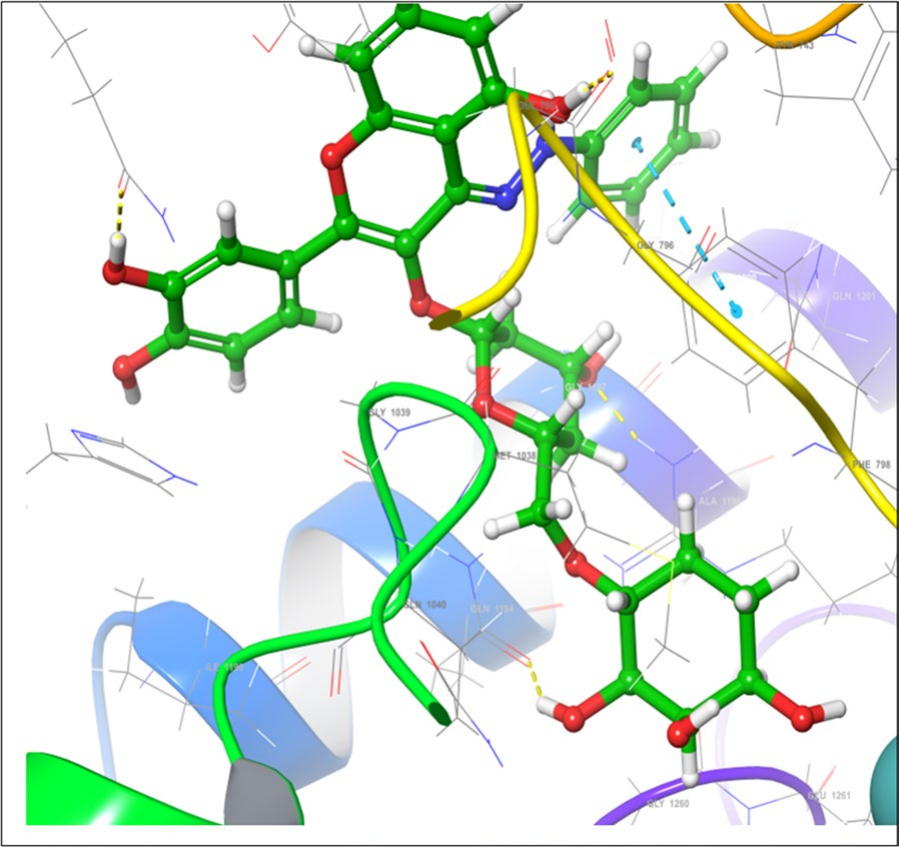
3D pose of RU3a_3_ inside the binding pocket

**Fig. 3:**
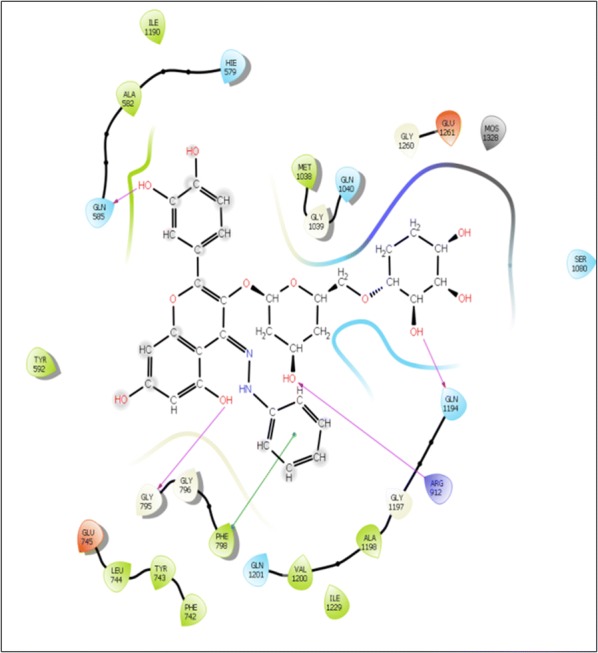
2D pose of RU3a_3_ inside the binding pocket

**Fig. 4:**
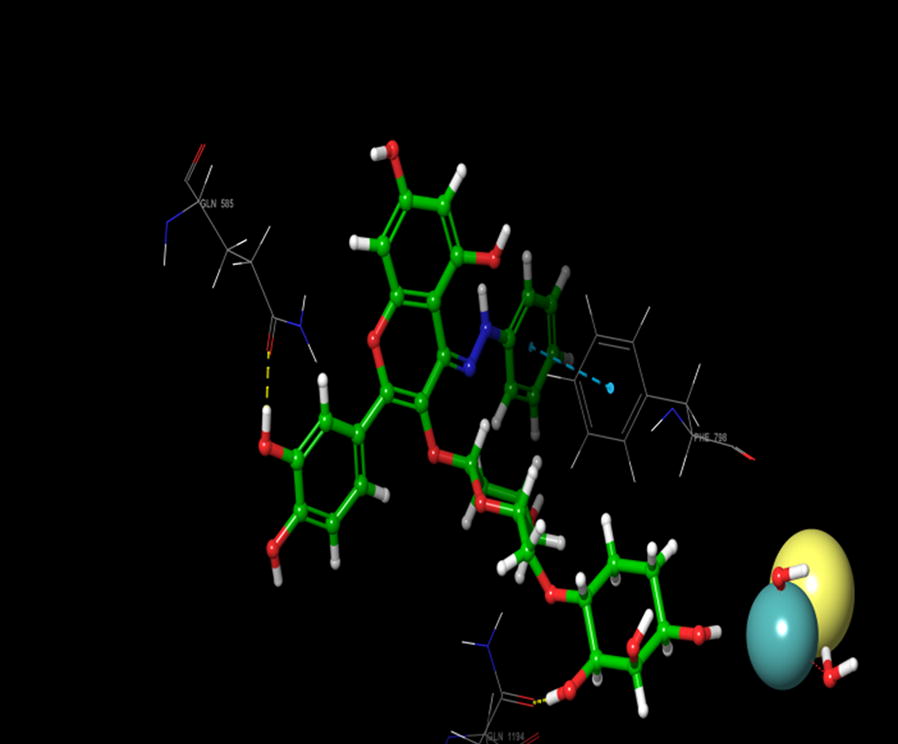
3D pose of RU3a_3_ showing hydrogen bonding (yellow) with GLN1194, ARG 912, GLY795, GLN 585 and π–π bonding (blue) with PHE798

Visual inspection of 3D poses of RU3a_3_ displayed a compact arrangement of polar and hydrophobic residues around the ligand forming a narrow passage in XO binding cavity with a docking score/binding score of − 13.244 and binding energy − 91.242 kJ/mol. An interesting pi–pi bonding was observed between benzene ring of phenyl hydrazine and hydrophobic residue PHE 798 of active site (Figs. 1, 2, 3). Along with this a strong hydrogen bonding was observed between OH group of rutinoside and polar residue GLN 1194 and negatively charged ARG 912 (Fig. 4). Similarly ARG 912 was found essential in the study of Shen et al. during the comparison of curcumin derivatives with quercetin and leuteolin [39]. Another hydrogen bonding was visualized between Chromene moiety and the residues of active site namely GLY 795 ad GLN585. Other hydrophobic amino acid residues closely placed within the cavity were observed as PHE 798, VAL1200, ALA1198, TYR 592, MET 1038 and ILE1229.

**Fig. 5:**
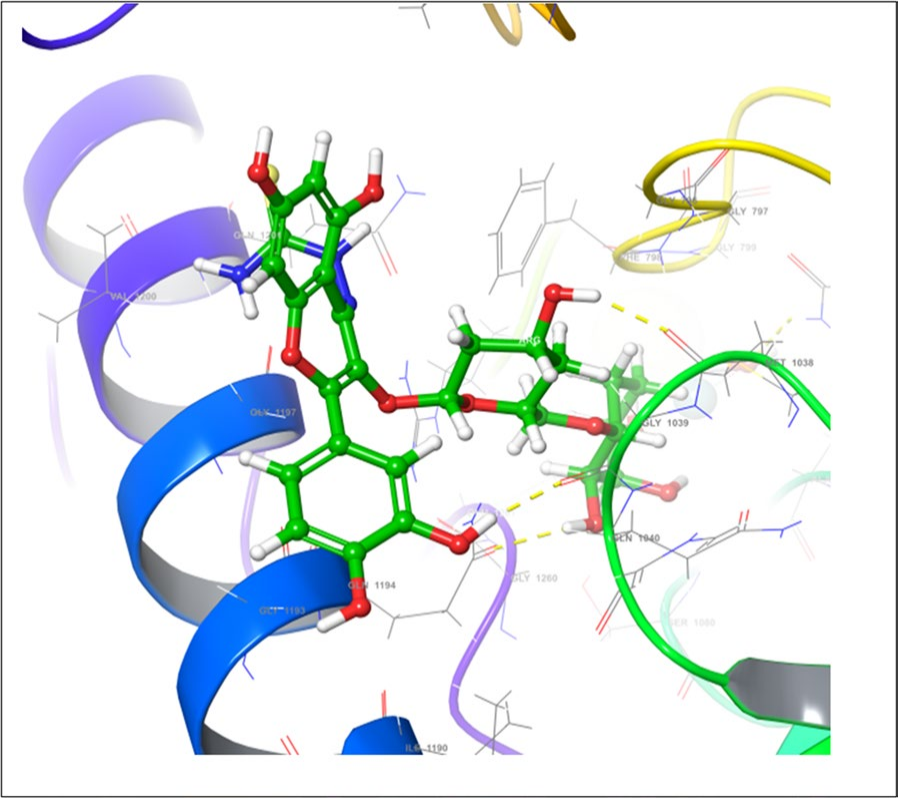
3D pose of RU3a_1_ inside the binding pocket

**Fig. 6:**
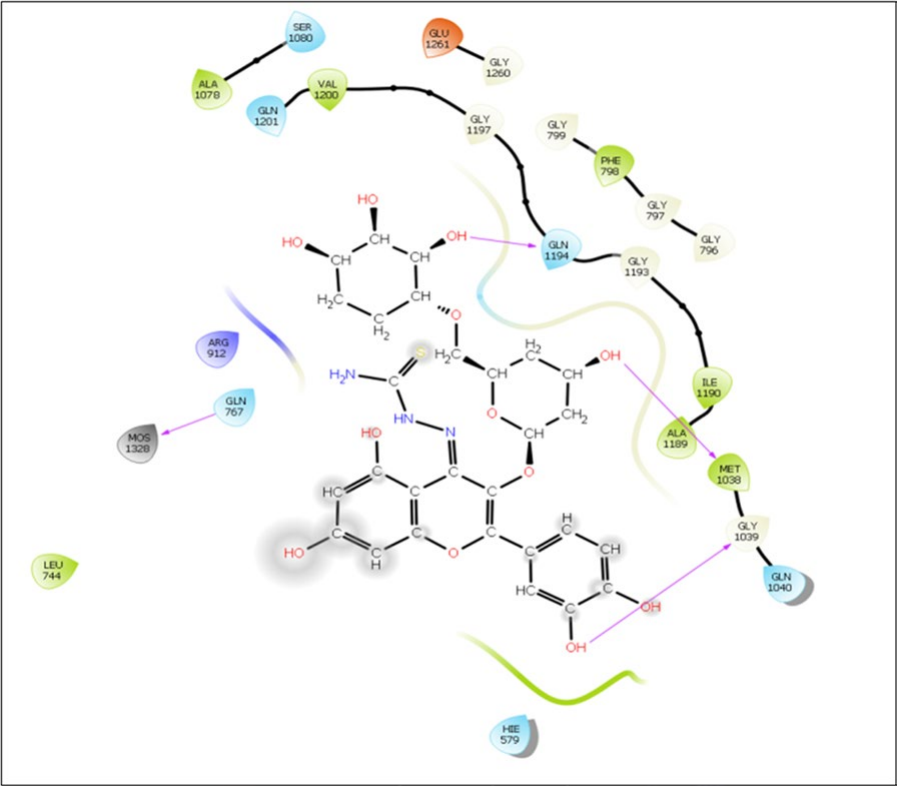
2D pose of RU3a_1_ inside the binding pocket

**Fig. 7:**
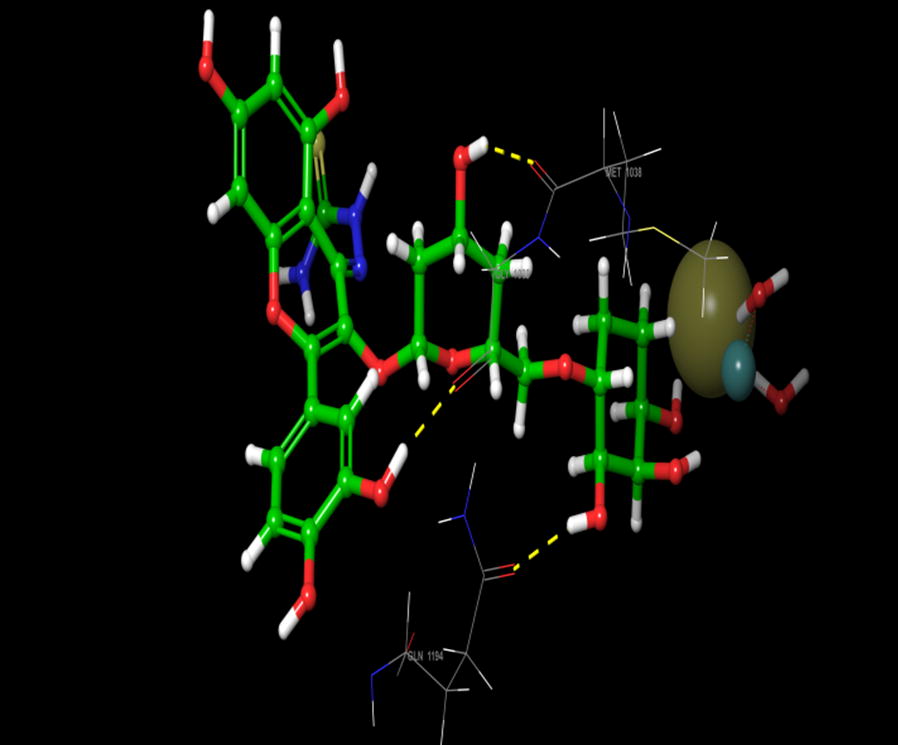
3D pose of RU3a_1_ showing hydrogen bonding with GLN 1194, MET1038 and GLY 1039

**Fig. 8:**
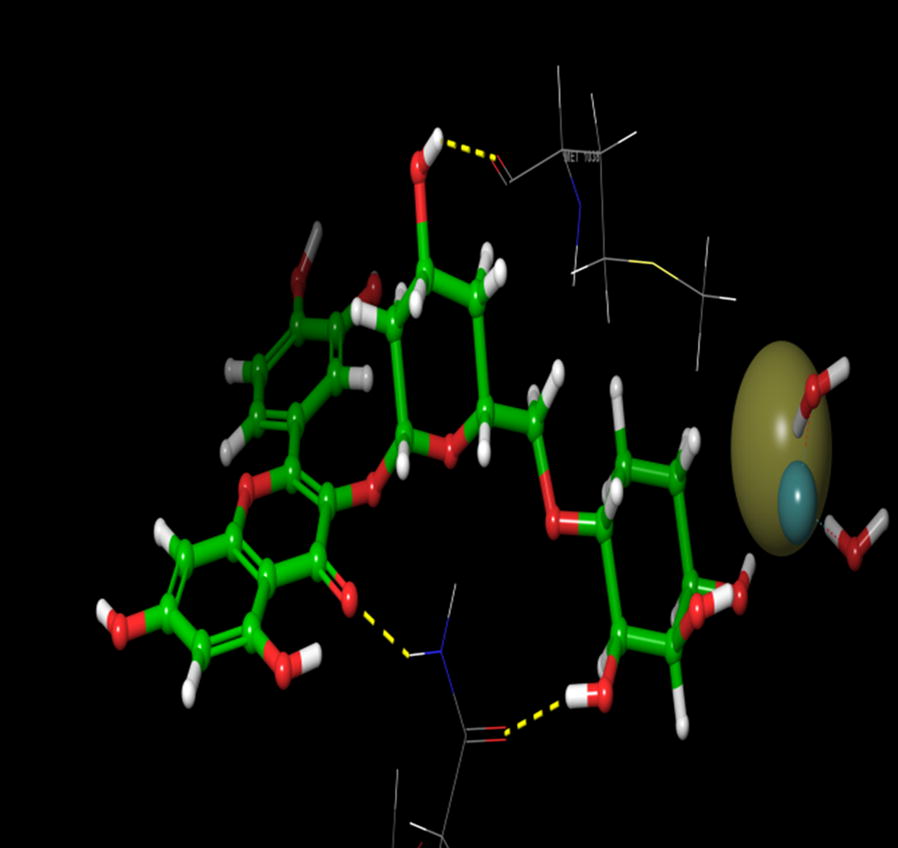
3D pose of rutin showing hydrogen bonding with GLN 1194 and MET1038

**Fig. 9:**
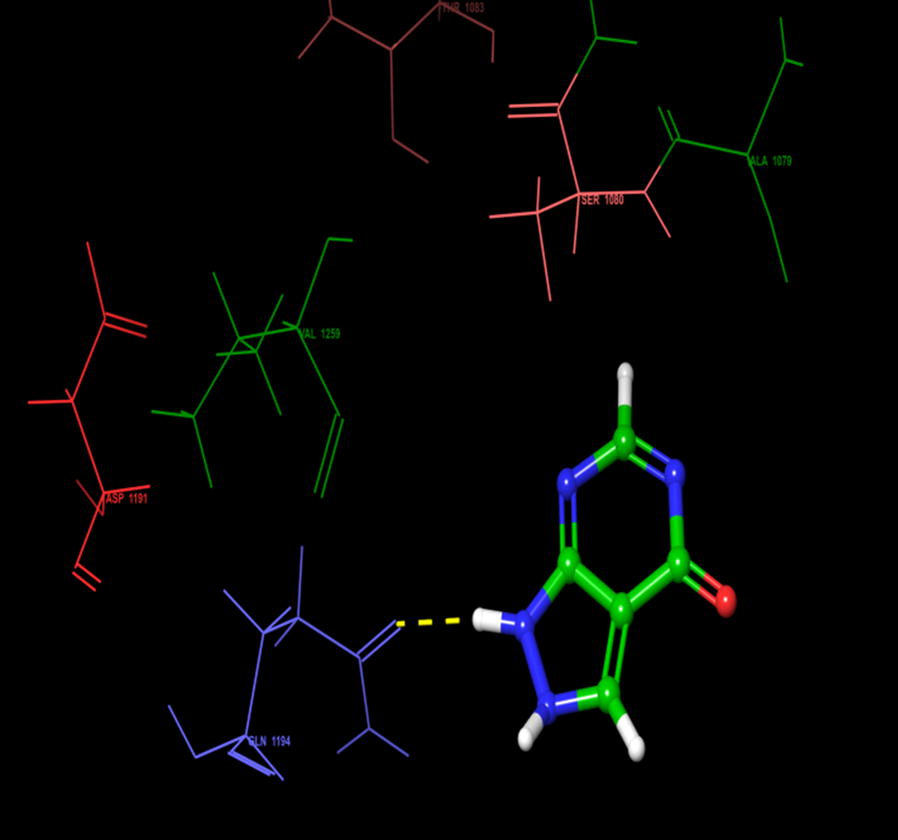
3D pose of allopurinol showing hydrogen bonding with GLN 1194

On the other hand, during the visualization of RU3a_1_ the hydrogen bond was observed with OH group of phenyl ring and hydrophobic residue MET 1038 (Figs. 5, 6). Another hydrogen bond was found similar to RU3a_3_ between OH group of rutinoside and polar residue GLN1194 (Fig. 7). One more hydrogen bonding was observed between one of the OH group of dihydroxyphenyl ring and GLY1039. One more interaction was observed with the surrounding residue GLN 767 which forms a hydrogen bond with MOS 1328 (molybdenum metal ion) forming a closed channel to prevent the entry of substrate in the binding site. Other residues surrounding the ligand were observed as ARG 912, HIE 579, GLU 1261, ALA 1189 and ILE1198. When the 3D poses of these two compounds were compared with the native rutin structure, GLN 1194 forms 2 H-bonds, one with the C=O group of rutin and another with OH group of rutinoside (Fig. 8). The amino acid residues GLU1261 and GLN 1194 were found to be interacted similarly in the study of verbascoside by Wan et al. [40]. Beside this one H-bond was formed between OH group of chromene ring and MET1038. No pi–pi interaction was in the native structure rutin. In case of Allopurinol, the active site residues surrounding ligand were almost similar and placed near to MOS 1328. The hydrogen bond was observed between purine ring of allopurinol and GLN1194 (Fig. 9).

### In-vitro xanthine oxidase inhibitory activity

In order to monitor the efficacy of different synthesized rutin derivatives, xanthine oxidase inhibitory activity was determined using xanthine oxidase activity assay kit purchased from Sigma-aldrich Co. Allopurinol (positive control) reported to inhibit xanthine oxidase was also screened under identical conditions for comparison. The inhibition ratios revealed the xanthine oxidase inhibitory activity of the synthesized rutin derivatives and the results were summarized in Table 3. As expected, these rutin derivatives exhibited remarkable activity comparable to the positive control. Based on the in vitro activity; it was observed that hydrazine (RU3a_1_–RU3a_3_) and anilline analogues (RU4b_1_–RU4b_2_) were considerably more effective than ester derivatives (RU7c_1_–RU7c_3_). All the compounds of hydrazine series (RU3a_1_–RU3a_3_) were effective with IC_50_-values ranging from 04.870 to 09.924 µM. Rutin hybridized with phenyl hydrazine demonstrated highest activity against xanthine oxidase. While thisemicarbazide and phenylthiosemicarbazide derivatives of rutin showed a slight decrease in activity indicating the role of sulfur group in diminishing the inhibition and NH–NH_2_ group in enhancing the activity of targeted enzyme. Surprisingly, substitution of NH–NH_2_ with NH_2_ group leads to decrease of inhibitory activity. Ester derivatives of rutin synthesized after the hydrolysis of rutin exhibited a weaker inhibition than the positive control Allopurinol.

**Table 3:** In vitro xanthine oxidase inhibitory activity of rutin derivatives

Compound	IC_50_ (µM) ± SEM	Compound	IC_50_ (µM) ± SEM
**Rutin**	20.867 ± 0.12	**RU4b**_**2**_	12.541 ± 0.45
**RU3a**_**1**_	09.924 ± 0.01	**RU7c**_**1**_	19.377 ± 0.38
**RU3a**_**2**_	07.905 ± 0.15	**RU7c**_**2**_	17.428 ± 0.01
**RU3a**_**3**_	04.870 ± 0.02	**RU7c**_**3**_	13.476 ± 0.25
**RU4b**_**1**_	15.037 ± 0.01	**Allopurinol**	10.410 ± 0.72

SEM, standard error of the mean

The results of in vitro activity showed 80% similarity with the results of molecular docking with a few exceptions. In concordance with the screening and output of molecular docking RU3a_3_ comes out to be most active rutin derivative showing very good interaction with xanthine oxidase at molecular level. Elimination of rutinoside from rutin to synthesize ester derivatives results in a loss of potency with a threefold decrease of inhibitory potential.

### Structure activity relationship (SAR)

**Fig. 10:**
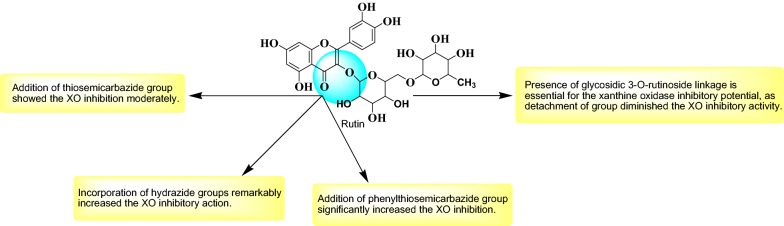
Structure activity relationship (SAR) of synthesized compounds

Few interesting notions about the relationship of activity and structures of synthesized compounds emerged from the present research (Fig. 10): (A) Rutinoside moiety seems to be important for the activity, as deletion of this leads to loss of activity could be seen from xanthine oxidase inhibitory activity Table 3. Which shows RU3a_3_ (Having rutinoside group) exhibited highest activity with an IC50 value 04.870 µM among all the compounds and RU7c_1_ showed lowest activity and fivefold decrease of activity with an IC_50_ value 19.377 µM. (B) Hydrazine derivatives were found to be more effective than the aniline derivatives revealing the importance of NH–NH_2_ group. But substitution of sulfur group along with hydrazines decreases the activity as in RU3a_3_ and RU3a_2_ and substitution of phenyl group along with sulfur improves the activity (RU3a_1_). (C) Substitution with ester group leads to a decrease of inhibitory activity.

### Enzyme kinetic analysis for XO-inhibitory activity

**Fig. 11:**
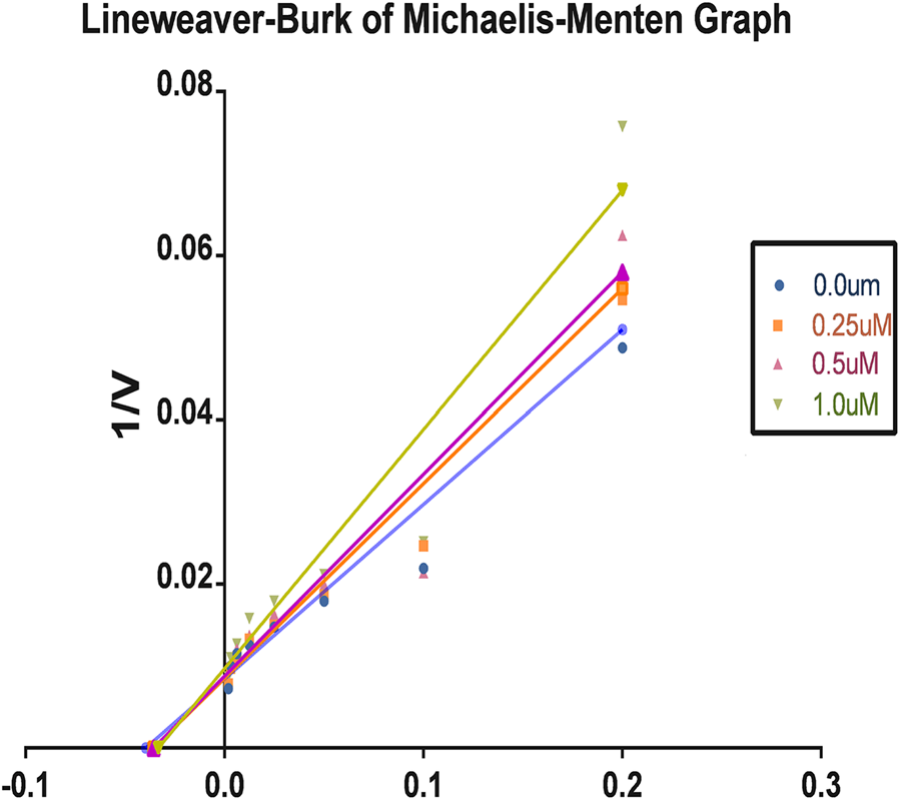
Lineweaver–Burk plot for RU3a_3_ against different concentrations

To determine the XO-inhibitory mechanisms of newly synthesized derivatives, we carried out kinetic studies of most active compound RU3a_3_ using Graph pad prism software. Firstly Michaelis–Menten curve was plotted for the enzyme activity at different concentrations of RU3a_3_ against different concentration of substrate (xanthine) Fig. 11.

**Fig. 12:**
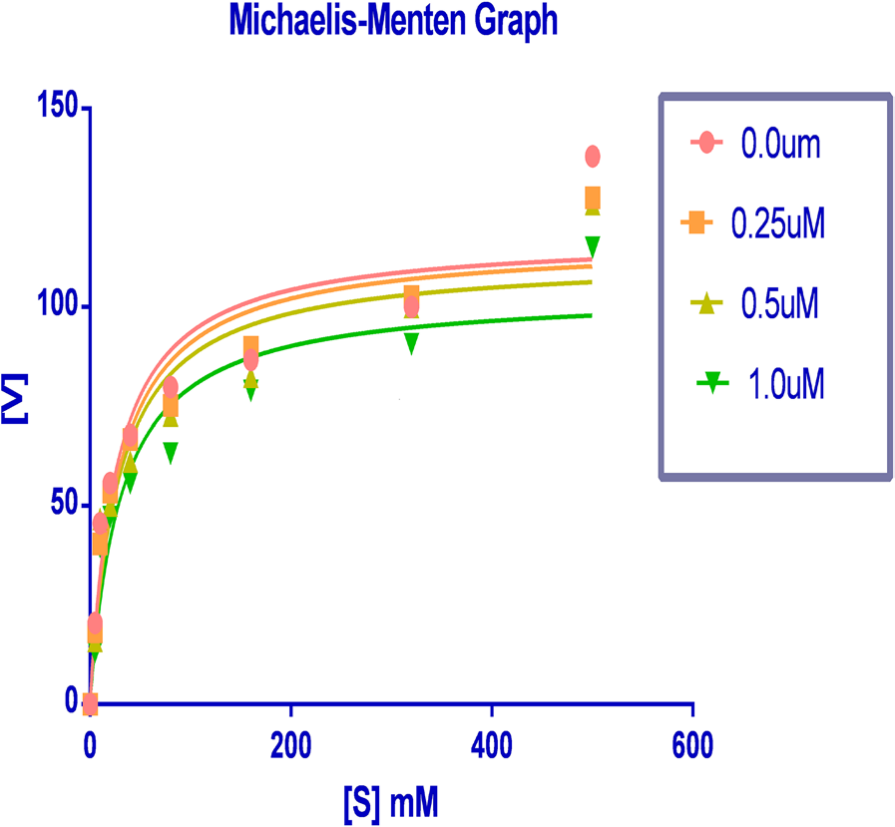
Michaelis–Menten curve for RU3a_3_ at different concentrations

Then double reciprocal plot (Lineweaver–Burk) analysis was done in the presence (0.25, 0.5, and 1.0 µM) and absence of RU3a_3_ from in vitro data generated during the oxidation of xanthine in presence of xanthine oxidase (Fig. 12). The x- and y axis intercepts of the Lineweaver–Burk plot were utilized to calculate K_m_ and V_max_ values of RU3a_3_ at different concentrations (Table 4).

**Table 4:** K_m_ and V_max_ values of xanthine oxidase at different concentrations of RU3a_3_

S. no.	Conc. of RU3a_3_ (µM)	Km (µM)	V_max_ (µmol/min)
1.	0.0	27.21	119.6
2.	0.25	30.11	114.4
3.	0.5	32.90	108.2
4.	1.0	35.08	98.7

A concentration-dependent decrease of V_max_ was predicted in contrast to K_m_ value which was found to increasing when concentration of RU3a_3_ was increased. The intersection of linear straight lines drawn against each concentration was located at same point, suggesting that RU3a_3_ reacts in competitive manner during the inhibition of xanthine oxidase.

### In-vitro evaluation of antioxidant activity by DPPH and H_2_O_2_ method

The antioxidant potential of newly synthesized compounds was evaluated by DPPH and Hydrogen peroxide radical assay. The comparative analysis of IC_50_ values for both the assays was done and the results were found to be impressive (Table 5). The results evinced a noteworthy inhibition of DPPH almost all the compounds when compared with the positive control ascorbic acid. In case of DPPH assay compound RU4b_1_ was demonstrated as most potent compound against oxidative stress caused because of free radicals having an IC_50_ value of 02.647 ± 0.09 µM. Along with this compound RU3a_1_ also showed very good antioxidant potential with an IC_50_ value of 05.021 ± 0.10 µM. When the detailed structure activity relationship was developed between these compounds, it was concluded that both the compounds having hydrazine linkage derived from phenyl hydrazine and phenyl thiosemicarbazide. Similarly, during the analysis of hydrogen peroxide assay all the compounds with hydrazines substitution showed very good antioxidant potential having IC_50_ in range of 04.146 ± 0.01 to 09.134 ± 0.35 (Fig. 7). Compound RU3a_2_ having phenyl thiosemicarbazide substitution showed potential antioxidant activity among all the derivatives. Along with this phenyl hydrazine substituted rutin derivative (RU3a_3_) also showed very good scavenging activity with an IC_50_ value of 06.561 ± 0.10. When the detailed structure activity relationship was developed between these compounds, it was concluded that both the compounds having hydrazine linkage derived from phenyl hydrazine and phenyl thiosemicarbazide.

**Table 5:** Antioxidant activity of synthesized derivatives by DPPH and H_2_O_2_ method

Compound	IC_50_ (µM) ± SEM	IC_50_ (µM) ± SEM
RU3a_1_	05.021 ± 0.10	09.134 ± 0.35
RU3a_2_	08.728 ± 0.02	04.146 ± 0.01
RU3a_3_	11.688 ± 0.01	06.561 ± 0.10
RU4b_1_	02.647 ± 0.09	09.863 ± 0.25
RU4b_2_	08.476 ± 0.25	04.378 ± 0.01
RU7c_1_	06.056 ± 0.13	14.731 ± 0.60
RU7c_2_	14.669 ± 0.01	12.126 ± 0.20
RU7c_3_	07.692 ± 0.42	17.884 ± 0.41
RU001	09.483 ± 0.08	18.623 ± 0.07
Ascorbic acid	22.195 ± 0.08	22.195 ± 0.08

SEM, standard error of the mean

## Conclusion

Starting from the structures of rutin as anti-XO hit previously identified, different series of novel analogues were designed and synthesized to explore the structure–activity relationships associated with these xanthine oxidase inhibitors along with their antioxidant potential. Different structural elements were identified as essential for antioxidant and anti-XO properties, such as the presence of rutinoside (RU3a_1_, RU3a_2_ and RU3a_3_) comes out as important skeleton for the inhibitory potential, presence of hydrazone linker along with phenyl group, while the associated xanthine oxidase inhibitory effect was found to follow a different trend for the two series hydrazine (RU3a_1–3_) and ester derivatives (RU7c_1–3_). The newly synthesized derivatives with anti-oxidant and ani-XO IC_50_ values in the low micromolar range and good selectivity indexes were identified. Contemporary synthetic efforts are focused towards the insertion of the hydrazones and ester linkage by replacing the side linkage rutinoside of rutin with more stable groups while maintaining the overall length of new derivatives. Molecular docking provide an improved trail to design the new molecules with an avantgarde stability and potency.

## Additional file

**Additional file 1.** HNMR spectra of compound RU3a_3_

## Data Availability

Not applicable.

## References

[1] Berry CE, Hare JM (2004). Xanthine oxidoreductase and cardiovascular disease: molecular mechanisms and pathophysiological implications. J Physiol.

[2] Moriwaki Y, Yamamoto T, Higashino K (1997). Distribution and pathophysiologic role of molybdenum-containing enzymes. Histol Histopathol.

[3] Klinenberg JR, Goldfinger SE, Seegmiller JE (1965). The effectiveness of the xanthine oxidase inhibitor allopurinol in the treatment of gout. Ann Intern Med.

[4] Yu KH (2007). Febuxostat: a novel non-purine selective inhibitor of xanthine oxidase for the treatment of hyperuricemia in gout. Recent Pat Inflamm Allergy Drug Discov.

[5] Battelli MG, Bolognesi A, Polito L (2014). Pathophysiology of circulating xanthine oxidoreductase: new emerging roles for a multi-tasking enzyme. Biochim Biophys Acta Mol Basis Dis.

[6] Brass CA, Narciso J, Gollan JL (1991). Enhanced activity of the free radical producing enzyme xanthine oxidase in hypoxic rat liver. Regulation and pathophysiologic significance. J Clin Invest.

[7] Chambers DE, Parks DA, Patterson G, Roy R, McCord JM, Yoshida S, Parmley LF, Downey JM (1985). Xanthine oxidase as a source of free radical damage in myocardial ischemia. J Mol Cell Cardiol.

[8] Desco MC, Asensi M, Márquez R, Martínez-Valls J, Vento M, Pallardó FV, Sastre J, Viña J (2002). Xanthine oxidase is involved in free radical production in type 1 diabetes: protection by allopurinol. Diabetes.

[9] Kuppusamy P, Zweier JL (1989). Characterization of free radical generation by xanthine oxidase. Evidence for hydroxyl radical generation. J Biol Chem.

[10] Dawson J, Walters M (2006). Uric acid and xanthine oxidase: future therapeutic targets in the prevention of cardiovascular disease?. Br J Clin Pharmacol.

[11] Khosla UM, Zharikov S, Finch JL, Nakagawa T, Roncal C, Mu W, Krotova K, Block ER, Prabhakar S, Johnson RJ (2005). Hyperuricemia induces endothelial dysfunction. Kidney Int.

[12] Kaynar H, Meral M, Turhan H, Keles M, Celik G, Akcay F (2005). Glutathione peroxidase, glutathione-S-transferase, catalase, xanthine oxidase, Cu–Zn superoxide dismutase activities, total glutathione, nitric oxide, and malondialdehyde levels in erythrocytes of patients with small cell and non-small cell lung cancer. Cancer Lett.

[13] Griguer CE, Oliva CR, Kelley EE, Giles GI, Lancaster JR, Gillespie GY (2006). Xanthine oxidase-dependent regulation of hypoxia-inducible factor in cancer cells. Cancer Res.

[14] Kanellis J, Kang DH (2005). Uric acid as a mediator of endothelial dysfunction, inflammation, and vascular disease. Seminars in nephrology.

[15] Miesel R, Zuber M (1993). Elevated levels of xanthine oxidase in serum of patients with inflammatory and autoimmune rheumatic diseases. Inflammation.

[16] Wijermars LG, Bakker JA, de Vries DK, van Noorden CJ, Bierau J, Kostidis S, Mayboroda OA, Tsikas D, Schaapherder AF, Lindeman JH (2016). The hypoxanthine–xanthine oxidase axis is not involved in the initial phase of clinical transplantation-related ischemia–reperfusion injury. Am J Physiol Renal Physiol.

[17] Poles MZ, Bódi N, Bagyánszki M, Fekete É, Mészáros AT, Varga G, Szűcs S, Nászai A, Kiss L, Kozlov AV, Boros M (2018). Reduction of nitrosative stress by methane: neuroprotection through xanthine oxidoreductase inhibition in a rat model of mesenteric ischemia–reperfusion. Free Radic Biol Med.

[18] Osada Y, Tsuchimoto M, Fukushima H, Takahashi K, Kondo S, Hasegawa M, Komoriya K (1993). Hypouricemic effect of the novel xanthine oxidase inhibitor, TEI-6720, in rodents. Eur J of Pharmacol.

[19] Krakoff IH, Meyer RL (1965). Prevention of hyperuricemia in leukemia and lymphoma: use of allopurinol, a xanthine oxidase inhibitor. JAMA.

[20] Pacher PA, Nivorozhkin A, Szabó C (2006). Therapeutic effects of xanthine oxidase inhibitors: renaissance half a century after the discovery of allopurinol. Pharmacol Rev.

[21] Inkster ME, Cotter MA, Cameron NE (2007). Treatment with the xanthine oxidase inhibitor, allopurinol, improves nerve and vascular function in diabetic rats. Eur J Pharmacol.

[22] Sagor M, Taher A, Tabassum N, Potol M, Alam M (2015). Xanthine oxidase inhibitor, allopurinol, prevented oxidative stress, fibrosis, and myocardial damage in isoproterenol induced aged rats. Oxid Med Cell Longev.

[23] Min HK, Lee B, Kwok SK, Ju JH, Kim WU, Park YM, Park SH (2015). Allopurinol hypersensitivity syndrome in patients with hematological malignancies: characteristics and clinical outcomes. Korean J Intern Med.

[24] Quach C, Galen BT (2018). HLA-B* 5801 testing to prevent allopurinol hypersensitivity syndrome: a teachable moment. JAMA Int Med.

[25] Takano Y, Hase-Aoki K, Horiuchi H, Zhao L, Kasahara Y, Kondo S, Becker MA (2005). Selectivity of febuxostat, a novel non-purine inhibitor of xanthine oxidase/xanthine dehydrogenase. Life Sci.

[26] Mayer MD, Khosravan R, Vernillet L, Wu JT, Joseph-Ridge N, Mulford DJ (2005). Pharmacokinetics and pharmacodynamics of febuxostat, a new non-purine selective inhibitor of xanthine oxidase in subjects with renal impairment. Am J Ther.

[27] Nepali K, Singh G, Turan A, Agarwal A, Sapra S, Kumar R, Banerjee UC, Verma PK, Satti NK, Gupta MK, Suri OP (2011). A rational approach for the design and synthesis of 1-acetyl-3, 5-diaryl-4, 5-dihydro (1H) pyrazoles as a new class of potential non-purine xanthine oxidase inhibitors. Bioorg Med Chem.

[28] Becker MA, Kisicki J, Khosravan R, Wu J, Mulford D, Hunt B, MacDonald P, Joseph-Ridge N (2004). Febuxostat (TMX-67), a novel, non-purine, selective inhibitor of xanthine oxidase, is safe and decreases serum urate in healthy volunteers. Nucleosides Nucleotides Nucleic Acids.

[29] Khosravan R, Grabowski BA, Wu JT, Joseph-Ridge N, Vernillet L (2006). Pharmacokinetics, pharmacodynamics and safety of febuxostat, a non-purine selective inhibitor of xanthine oxidase, in a dose escalation study in healthy subjects. Clin Pharmacokinet.

[30] Malik N, Dhiman P, Khatkar A (2017). In-silico design and ADMET studies of natural compounds as inhibitors of xanthine oxidase (XO) enzyme. Curr Drug Metab.

[31] Muhammad A, Arthur DE, Babangida S, Erukainure OL, Malami I, Sani H, Abdulhamid AW, Ajiboye IO, Saka AA, Hamza NM, Asema S (2018). Modulatory role of rutin on 2, 5-hexanedione-induced chromosomal and DNA damage in rats: validation of computational predictions. Drug Chem Toxicol.

[32] Roleira FM, Varela CL, Costa SC, Tavares-da-Silva EJ (2018). Phenolic derivatives from medicinal herbs and plant extracts: anticancer effects and synthetic approaches to modulate biological activity. Nat Prod Chem.

[33] Baldisserotto A, Vertuani S, Bino A, De Lucia D, Lampronti I, Milani R, Gambari R, Manfredini S (2015). Design, synthesis and biological activity of a novel Rutin analogue with improved lipid soluble properties. Bioorg Med Chem.

[34] Gullon B, Lú-Chau TA, Moreira MT, Lema JM, Eibes G (2017). Rutin: a review on extraction, identification and purification methods, biological activities and approaches to enhance its bioavailability. Trends Food Sci Technol.

[35] Friesner RA, Banks JL, Murphy RB, Halgren TA, Klicic JJ, Mainz DT, Repasky MP, Knoll EH, Shelley M, Perry JK, Shaw DE (2004). Glide: a new approach for rapid, accurate docking and scoring. 1. Method and assessment of docking accuracy. J Med Chem.

[36] Dhiman P, Malik N, Verma PK, Khatkar A (2015). Synthesis and biological evaluation of thiazolo and imidazo *N*-(4-nitrophenyl)-7-methyl-5-aryl-pyrimidine-6 carboxamide derivatives. Res Chem Intermed.

[37] Patel A, Patel A, Patel A, Patel NM (2010). Determination of polyphenols and free radical scavenging activity of *Tephrosia purpurea* linn leaves (Leguminosae). Pharmacogn Res.

[38] Li P, Tian Y, Zhai H, Deng F, Xie M, Zhang X (2013). Study on the activity of non-purine xanthine oxidase inhibitor by 3D-QSAR modeling and molecular docking. J Mol Struct.

[39] Shen L, Ji HF (2009). Insights into the inhibition of xanthine oxidase by curcumin. Bioorg Med Chem Lett.

[40] Wan Y, Zou B, Zeng H, Zhang L, Chen M, Fu G (2016). Inhibitory effect of verbascoside on xanthine oxidase activity. Int J Biol Macromol.

