# Supplementary figures and images for: In silico design and synthesis of targeted rutin derivatives as xanthine oxidase inhibitors
Source: BMC Chem. 2019 May 23;13(1):71. doi: 10.1186/s13065-019-0585-8 (PMC6661775; doi:10.1186/s13065-019-0585-8)

—10.13

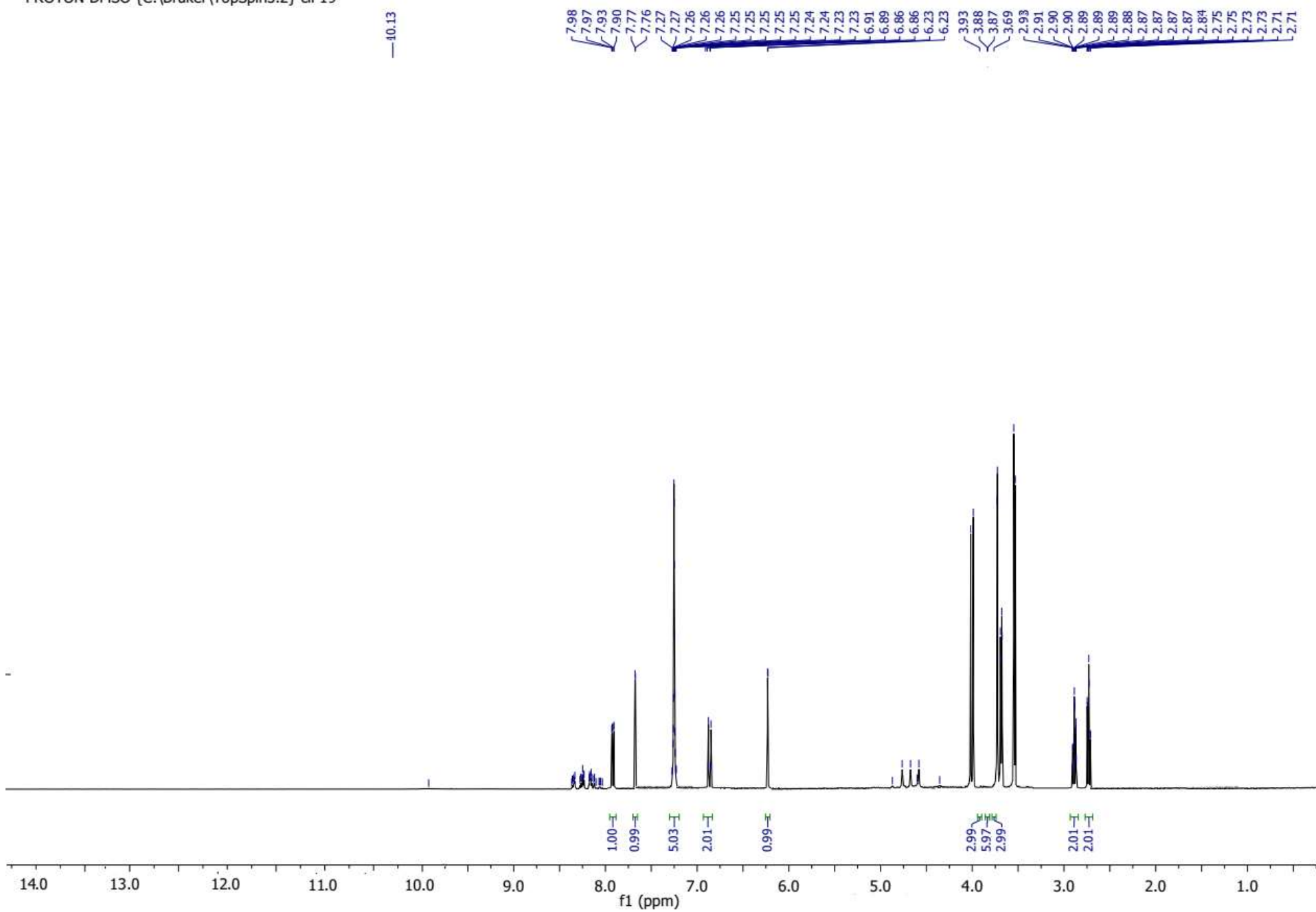

Supplement: Supplementary file 1 — Additional file 1. HNMR spectra of compound RU3a3 [file 13065_2019_585_MOESM1_ESM.pdf]
